# Human fibroblasts display a differential focal adhesion phenotype relative to chimpanzee

**DOI:** 10.1093/emph/eow010

**Published:** 2016-03-12

**Authors:** Alexander S. Advani, Annie Y. Chen, Courtney C. Babbitt

**Affiliations:** ^1^Department of Biology, Duke University, Durham, NC 27708, USA; ^2^Department of Biology, University of Massachusetts Amherst, Amherst, MA 01003, USA

**Keywords:** human evolution, focal adhesion, fibroblast, cancer

## Abstract

There are a number of documented differences between humans and our closest relatives in responses to wound healing and in disease susceptibilities, suggesting a differential cellular response to certain environmental factors. In this study, we sought to look at a specific cell type, fibroblasts, to examine differences in cellular adhesion between humans and chimpanzees in visualized cells and in gene expression. We have found significant differences in the number of focal adhesions between primary human and chimpanzee fibroblasts. Additionally, we see that adhesion related gene ontology categories are some of the most differentially expressed between human and chimpanzee in normal fibroblast cells. These results suggest that human and chimpanzee fibroblasts may have somewhat different adhesive properties, which could play a role in differential disease phenotypes and responses to external factors.

## Abstract (precis)

It has been documented that there are differences in disease susceptibilities between humans and non-human primates. We investigate one of these differences in fibroblasts to examine differences in cellular adhesion between humans and chimpanzees using microscopy and gene expression and have found significant differences in both datasets. These results suggest that human and chimpanzee fibroblasts may have somewhat different adhesive properties, which could play a role in differential disease phenotypes and responses to external factors.

## BACKGROUND

Recent efforts to expose genotypic differences between humans and our closest relatives have uncovered the question of gene expression changes and the role they play in influencing phenotype. By determining the genetic differences between humans and chimpanzees, we can learn about how we have evolved and adapted during the ∼6 million year divergence between these species. Comparative analyses can be informative about not only evolution but also in questions of different disease susceptibilities between species [1, 2]. There have been a number of studies looking at differences in gene expression between humans and chimpanzees [3–7]; however, very few studies have undertaken a comparative analysis at the level of phenotypic cell biology in these species.

Our study uses focal adhesions to illustrate how we differ from our closest relatives at the phenotypic level, and how this relates to differences in gene expression. Focal adhesions are large complexes of proteins that are usually found at the periphery of cells [8]. One of their primary functions is to facilitate cell attachment to the extra-cellular matrix (ECM), primarily through the use of proteins called integrins [8, 9]. However, focal adhesions are also involved in several other aspects of cell function including cell–cell signaling, detection of the ECM and cell movement [8–10]. Focal adhesions play an early and vital role in many signaling pathways and allow cells to respond to various stimuli [8, 9, 11–13]. In addition, focal adhesions are key factors in cell mobility as they are transported from the lagging areas of a moving cell to its leading edge in order to form a new attachment with the ECM and stabilize the cell [8, 9, 14–17]. As a result, focal adhesions are critical to normal cell function as well as a cell’s ability to react to its environment [10, 16, 18, 19].

A phenotypic difference that affects focal adhesions could impair or modify basic cellular functions; interfering with focal adhesion function can cause reduced cell motility [10] and dramatic changes can affect the phenotype to the point of cell death [15, 16, 18–20]. In addition, differences in the prevalence and availability of many of the signaling proteins associated with focal adhesions have been shown to produce phenotypes such as cardiac disease or several types of cancer [21–23], and when some of these same proteins are targeted, they can have beneficial effects in the treatment of those diseases [13, 24]. Furthermore, there are broader implications to modifications in focal adhesions as a new phenotype can have an effect at the organismal level. For example, adjusting focal adhesion phenotype could cause differences in cell sensitivity due to increased signaling or faster wound healing due to increased cell motility [8, 9]. A significant difference in focal adhesion phenotype between species could be an indicator of a change in fibroblast adherence and interaction with the environment.

In order to assay focal adhesion phenotypes, a way of determining focal adhesion location and size is required. Focal adhesions are composed of several proteins and compounds, one of which is vinculin. Vinculin is a focal adhesion-specific protein that is critical to the structure of focal adhesions [20, 25, 26]. It is primarily involved in cell attachment and motility and localizes to focal adhesions [14, 27]. It is a key protein in cell structure as it anchors F-actin to the cell membrane at focal adhesions and has no close relatives that can fulfill its function if it is not present [28, 29]. Vinculin knockout mice were shown to die in early development and have defects in their partially formed hearts and brains [28]. Vinculin can be used as a proxy for focal adhesion presence as it is required for focal adhesion function, it is specific to focal adhesions and it is irreplaceable [29, 30]. Additionally, vinculin is pervasive throughout the focal adhesions’ structures, which allow it to be used to observe their size as well [30, 31]. As such it has been used by several studies to study focal adhesion location and function [29, 30]. Here, we examine focal adhesions within human and chimpanzee primary skin fibroblasts using this protein proxy. As a primarily exploratory study, we are investigating the following question: To what extent do human and chimpanzee skin fibroblasts differ in adhesive properties?

## METHODOLOGY

### Fibroblast staining

We examined vinculin antibody staining and cell size between representative fibroblast cell lines of the two species. Four human and four chimpanzee primary fibroblast cell lines (Coriell, Camden, NJ) were used for the experiment described here. All of the cell lines are from males of approximately the same age in each group (humans aged 22–30 years, chimpanzees aged 17–24 years) (Supplementary Table S1). Most of the human samples are from forearm samples, with one from the abdomen. Previous studies have shown that the area of the body sampled is important to note, with the upper and lower half of the body showing somewhat different changes in gene expression [32, 33]. For the chimpanzee lines, the area biopsied was not recorded by Yerkes Regional Primate Research Center although their protocols state that they should be from the ear pinna [5]. However, Shibata *et al.* [5] used similar cell lines in a gene expression and open chromatin analysis, and found that the fibroblast cells showed very little (<3%) overlap with the regionally affected genes from Rinn *et al.* [32], suggesting that the human and chimpanzee cells are from comparable regions of the body.

The fibroblast cells were expanded in Minimal Essential Medium with 10% fetal bovine serum until they were approximately 80% confluent. All cell lines were expanded until they were at similar population doubling levels and passage numbers. Cells were grown on glass coverslips in six-well plates for 24 hours (50 000 cells per well), and then fixed with a 4% formaldehyde solution. Following this, the cells underwent 3× washes with PBS before being permeabilized with 0.2% Triton-X100. The cells were then stained with a monoclonal anti-vinculin antibody (ABfinity; Thermo Fisher, Waltham, MA) and an AlexaFluor secondary (Molecular Probes; Thermo Fisher, Waltham, MA), with 3% BSA in PBS.

Cells were imaged using a Zeiss Axio Observer wide-field fluorescence microscope at Duke University’s Light Microscopy Core Facility. All cells that met the following criteria were imaged. The ∼10 clearest images from each cell line were then selected for further analysis giving a total of 60 images. Previous studies have normally had a total sample size of between 7 and 30 fibroblasts [34, 35]. The images were analyzed using the program MetaMorph. The size of the cell was determined by the actin staining. The actin image was then used to create a mask in order to determine the number and size of the focal adhesions on the exterior of the cell.

For the gene expression ontology enrichments, the raw data counts (generated using DGE-Seq [36]) were acquired from Shibata *et al.* [5], and normalization and differential expression were determined using edgeR [37]. These data were also generated from primary skin fibroblast lines and come from the same repository collections (Coriell, Camden, NJ), but are not from the same individual as the lines used in the analysis above. The categorical enrichments were performed using both custom software using the gene ontology (GO) databases [38].

## RESULTS AND DISCUSSION

### Experimental validation of differences in cell adhesion

**Figure 1.**
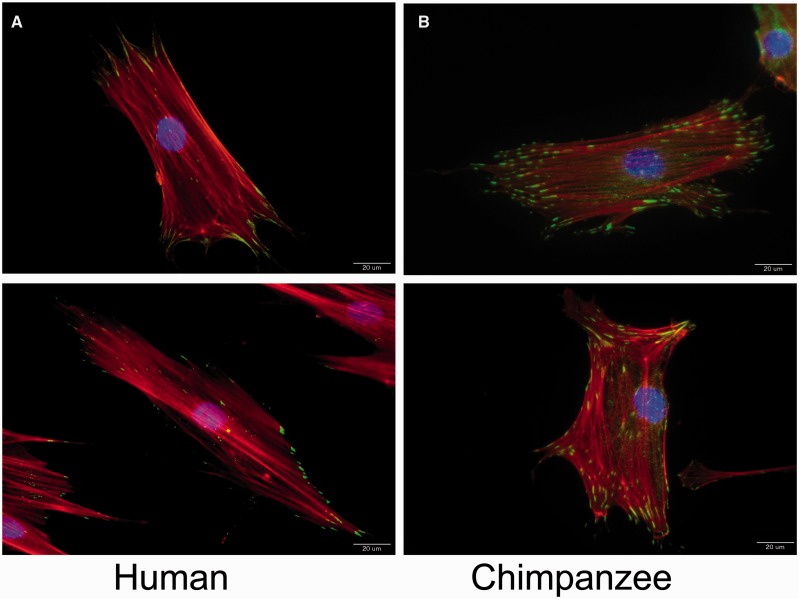
Example images of the stained human and chimpanzee fibroblast cells. Nuclei (blue), actin (red), and vinculin, a label for focal adhesions (green), are merged in these images, with 20 μm for the scale bar

**Figure 2.**
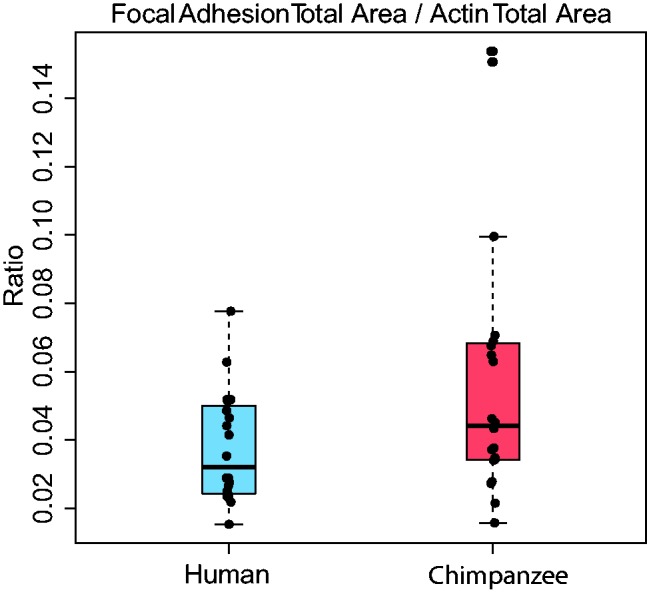
Box plot of the differences in the ratio of focal adhesion to total cell size (measured by actin staining). The human ratio is in blue and the chimpanzee in red

In order to begin to explore cell adhesion at a cellular level, we examined the differences in aspect of adhesion in human and chimpanzee primary fibroblast cells (Fig. 1). Vinculin can be used as a proxy indicator for focal adhesions, allowing for the detection of the number, size and location of the focal adhesions present within a cell [25, 26]. Vinculin is a focal adhesion-specific protein that is critical to the structure of focal adhesions [25, 26]. Additionally, transfection of vinculin cDNA into tumor cell lines expressing lower levels of the endogenous protein results in a significant suppression of their tumorigenic ability and an increase in substrate adhesiveness [39], suggesting that vinculin expression drives this change in phenotype. Here, we see that the ratio of focal adhesions to cell size between humans and chimpanzees is significantly different between species (Fig. 1), with an upregulation in the chimpanzee cells (analysis of variance, *P* < 0.00001). This is true even when we normalized for any differences in cell size, as measured by the actin cytoskeleton (Fig. 2). When the chimpanzee cell line with some outlier points is removed (S008975), this difference is also significant (*P* = 0.01476) (Supplementary Figure S1). The variance in the chimpanzee cell lines is much larger when cell lines are plotted individually (Supplementary Figure S1). Larger sample sizes will be needed in the future to determine more quantitatively what component of this variance is due to cell line or species effects. Given that the principal function of a focal adhesion is cell attachment [8], it is possible that the biological interpretation of this difference results in differential adhesive and migratory properties. It is possible that chimpanzee fibroblasts can attach to their substrate more firmly than human cells, which could indicate a difference in the way fibroblast cells move normally or in response to injury.

In the event of a disruption in tissue continuity, i.e. a wound, new cells must differentiate to replace those lost and healthy cells must migrate to seal the gaps in the tissue layer [40]. This results in a large number of focal adhesions being disassembled and reassembled at the leading edge of a migrating cell [40, 41]. Recent work has shown that the rate of wound closure will increase with a firmer wound bed [42]. Cells that are more firmly attached to their substrate might be more difficult to dislodge and may form a more robust cell layer. The efficiency with which a wound can be sealed and repaired could therefore be dramatically affected by the number of focal adhesions present in the cells forming the bed of the wound have. There have been reports of faster wound healing in wild primates than in humans [43, 44], but so far these are isolated events. Additionally, it is well documented that the gene expression response to wound healing is highly similar to gene expression changes in cancer progression [45, 46]. Epithelial cancers occur at much higher rates in humans than in non-human primates [47–50], and while most of that difference is likely due to environmental factors, there is evidence for some genetic and cellular differences as well.

We then looked at differences between human and chimpanzee fibroblasts at the level of gene expression based on the published gene expression dataset described Shibata *et al.* [5], which were also generated from primary skin fibroblast cell lines. We re-analyzed those differential gene expression data and found that two of the highest categories are related to cellular adhesive properties (biological adhesion, FDR = 1.07E−06 and cell adhesion, FDR = 1.84E−06) (Table 1 and Supplementary Table S2). Overall, categories involved in cellular adhesion are some of the most differentially expressed GO categories in human and chimpanzee fibroblasts (Table 1 and Supplementary Table S2).

**Table 1.** Top 10 GO biological process ontology enrichments from the fibroblast RNA-Seq data in Shibata *et al.* [5]

Category	*P* value	Total occurrence
Multicellular organismal process	7.36E−09	1048
Biological adhesion	7.78E−07	409
Cell adhesion	7.78E−07	409
Oxidation reduction	9.32E−06	339
Regulation of multicellular organismal process	2.94E−05	358
Developmental process	3.21E−05	1756
Cellular ion homeostasis	4.49E−05	117
Branching morphogenesis of a tube	5.10E−05	40
Morphogenesis of a branching structure	5.84E−05	41
Cellular chemical homeostasis	9.57E−05	119

The ontology enrichments were performed using the DAVID Functional Annotation tool [52], and used all genes measured in the study as the background gene set.

Our results suggest that the chimpanzee fibroblast cells might be naturally more adhesive, and the human cells then possibly more prone to changes towards cancer morphologies. These results illustrate the importance of comparative studies at the cellular level. Other studies looking at cellular differences in fibroblasts have also shown differential apoptotic function in humans when compared with chimpanzees [51], also suggesting that these species-specific differences in fibroblasts may lead to differences in epithelial cancers. It will be important to understand how these cellular phenotypes functionally link changes from gene expression to organismal phenotype, and how that might assist in our understanding of susceptibilities to diseases, such as epithelial cancer.

## CONCLUSION

Fibroblasts show differences in focal adhesions between humans and chimpanzees at both the genetic and cellular levels. Understanding the evolutionary history and phenotypic impact of these changes is essential in understanding the differences in fibroblast function in normal and diseased tissue. Our results suggest that human and chimpanzee fibroblasts may differ in adhesive properties, which then may play a role in differential phenotypes and responses to environmental factors.

## Supplementary Material

Supplementary Data
