# Supplementary material for: Human fibroblasts display a differential focal adhesion phenotype relative to chimpanzee
Source: Evol Med Public Health. 2016 Mar 12;2016(1):110–6. doi: 10.1093/emph/eow010 (PMC4804348; doi:10.1093/emph/eow010)
Supplement: Supplementary Data [file supp_2016_1_110__index.html]

Human Fibroblasts Display A Differential Focal Adhesion Phenotype Relative to Chimpanzee — Human fibroblasts display a differential focal adhesion phenotype relative to chimpanzee — Supplementary Data 

# Human fibroblasts display a differential focal adhesion phenotype relative to chimpanzee

## Supplementary Data

files

- Supplementary Data - pdf file
- Supplementary Data - xlsx file
- Supplementary Data - xlsx file
