# Supplementary figures and images for: Human fibroblasts display a differential focal adhesion phenotype relative to chimpanzee
Source: Evol Med Public Health. 2016 Mar 12;2016(1):110–6. doi: 10.1093/emph/eow010 (PMC4804348; doi:10.1093/emph/eow010)

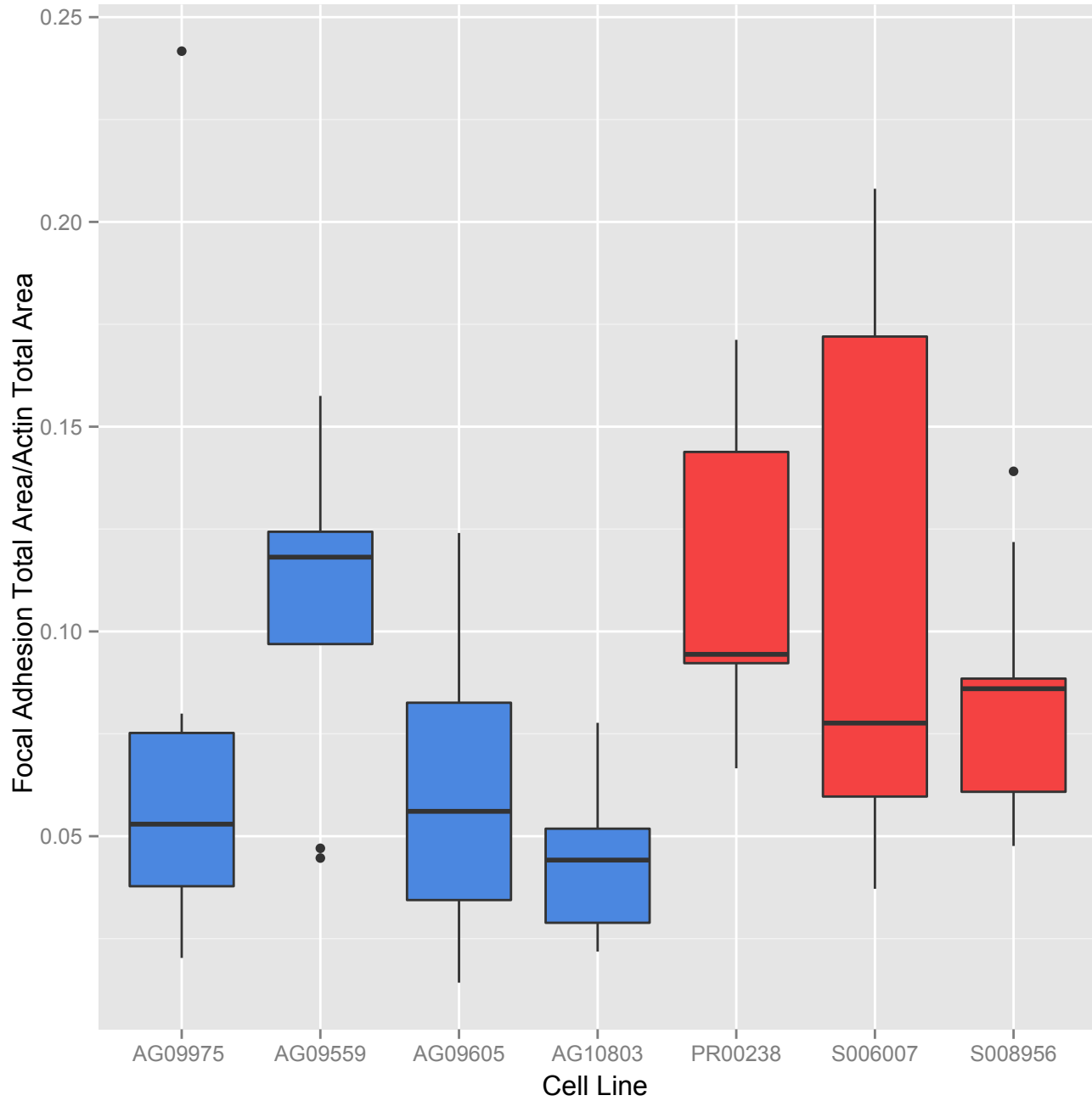

Supplement: Supplementary Data [file supp_eow010_FigureS1.pdf]
